# The Periodontal Benefit of Orthodontic Tooth Movement in a Deep Facial Recession of a Mandibular Incisor

**DOI:** 10.1155/2019/7601475

**Published:** 2019-10-20

**Authors:** Ivan Pedro Taffarel, Caio Seiti Miyoshi, Ivan Toshio Maruo, Thiago Martins Meira, Orlando Motohiro Tanaka

**Affiliations:** ^1^School of Life Sciences, Pontifícia Universidade Católica do Paraná, Curitiba, Brazil; ^2^Bahia State University (UNEB), Guanambi, Bahia, Brazil

## Abstract

Gingival recession refers to the exposure of the tooth's surface by an apical shift of the gingiva. The aim of this paper is to present a clinical case of an adult patient with a skeletal Class I and clinically deep gingival recession in the mandibular left central incisor. A preadjusted appliance with 0.022 in × 0.028 in slot was placed in both arches. Rectangular arches were used, with the addition of root lingual torque, specifically in the left lower central incisor. Class II and vertical intermaxillary elastics were used throughout the active treatment to obtain intercuspation of the posterior teeth. The orthodontic movement with the application of a localized biomechanics system of forces into the mandibular left central incisor delivered good dental and functional occlusion and, mainly, gingival and periodontal health. The follow-up showed stable results with the periodontium within normal limits and an improved occlusal interdigitation.

## 1. Introduction

Gingival recession refers to the exposure of the tooth's surface by an apical shift of the gingiva [1]. Numerous factors play roles in the development of gingival recession, like periodontal disease [2] and mechanical trauma [3], which are considered the primary factors in the pathogenesis of gingival recession. Although orthodontic treatment can lead to gingival recession by inaccurate performance [4], the etiology is often multifactorial [5].

Although there is controversy in the literature as to whether it is possible to have spontaneous improvement in gingival recession after orthodontic movement [6], recession can lead to poor esthetics [7] and tooth hypersensitivity [8].

The relationship between tooth movement by orthodontic treatment and the improvement of recession must be discussed because it has just been related in the literature to one of the multiple factors of recession etiology. Thus, the objective of this article is to present the clinical case of a patient who sought orthodontic treatment with the lower incisor showing localized and severe recession and a specific application of biomechanics with good results.

## 2. Diagnosis and Etiology

A female patient at the age of 21 years and 6 months sought orthodontic retreatment by appointment with her dentist who was concerned with the gingival recession of the left lower central incisor and with the indication of a gingival graft.

**Figure 1:**
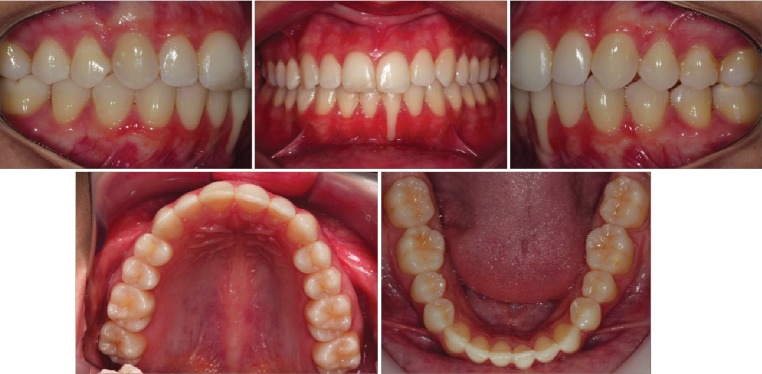
Pretreatment intraoral photographs.

In the dental aspect, she presented Class II, division 1, left subdivision malocclusion, 2 mm of overjet and 30% of overbite, presence of all permanent teeth, except the 3rd molars, good profile, general resorption of alveolar bone ridges, discrete periapical bone thinning in the apex, and clinically deep gingival recession in the left lower central incisor. It was possible to note that this tooth was slightly more extruded than the other teeth, with interference of the lower lip frena in the most apical region of the gingival recession. The moderate curve of Spee and parabolic dental arch form. The maxillary midline deviation was 1 mm to the right (Figure 1).

**Figure 2:**
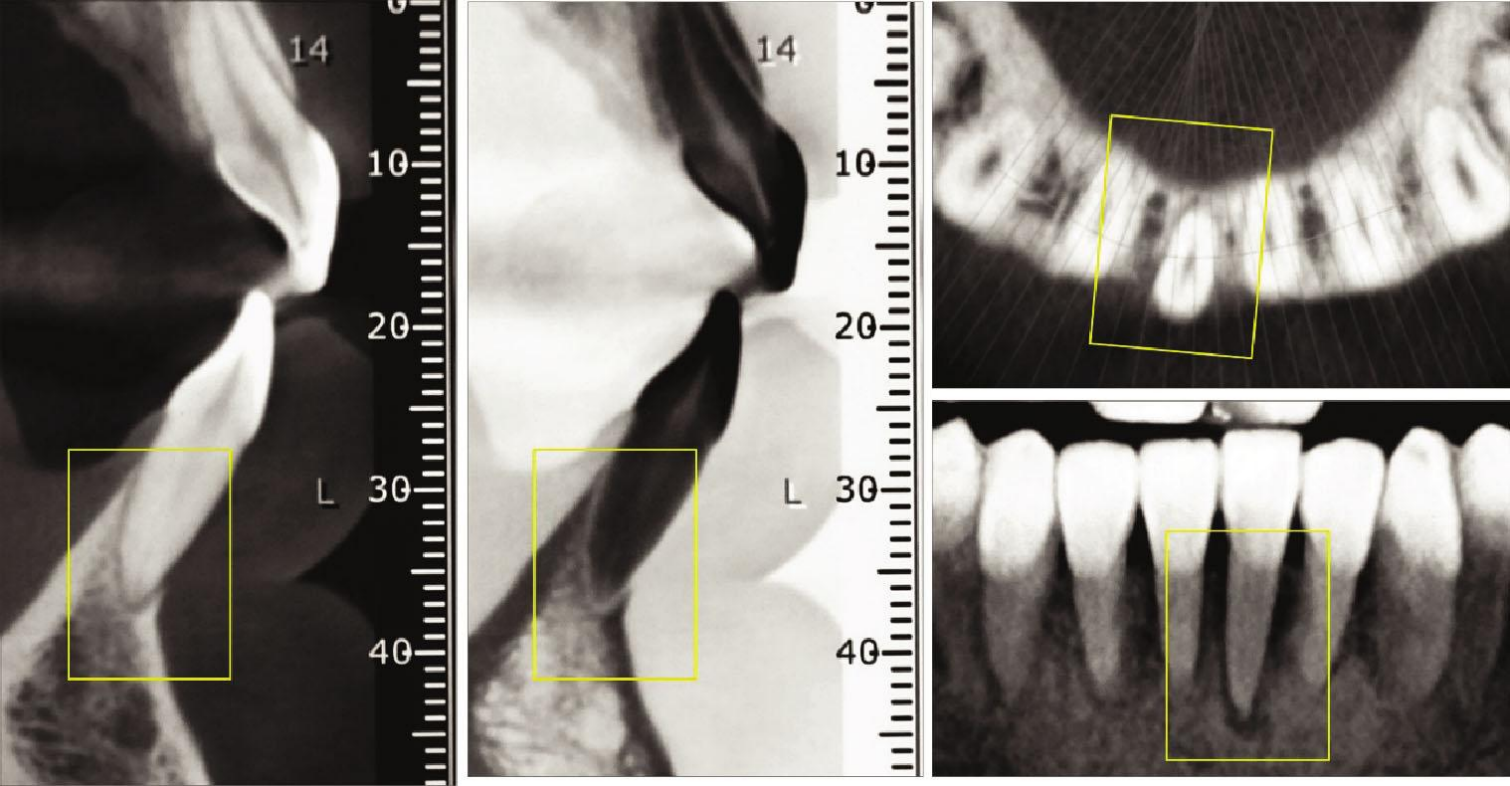
Initial CBCT of the left mandibular central incisor. Sagittal, coronal, and axial views.

The tomography showed no facial cortical bone of the left mandibular central incisor in the apical thirds of the root, with a slight hypodensity in the apical region. Lingual, mesial, and distal cortical bone of the central incisor was maintained (Figure 2).

## 3. Treatment Objectives

The following objectives of the orthodontic treatment were established: (1) to move the root of the inferior central incisor left to lingual; (2) wait for physiological coverage of the vestibular root face of the same center; and (3) obtain Class I of canines and molars in the left hemiarch.

## 4. Treatment Alternatives

Alternative 1: start with orthodontic treatment: aligning, leveling, lingual root torque in the left lower central incisor, and frenectomy. In the maxilla, to obtain intercuspation of the teeth of the left side moving all distally, TADs were used to optimize such movement. Gingival grafting was performed on the labial surface of the left lower central incisor after orthodontic completion.

Alternative 2: the same biomechanical procedure for the movement of the left lower incisor, however, keeping the left teeth side as they are, in Class II molar relationship.

## 5. Treatment Progress

Before the orthodontic treatment was started, the patient was referred to her general practitioner and the periodontist for routine clinical procedures. All treatment alternatives were explained to the patient, and alternative #1 was selected. It was explained, and she understood the little predictability in the reversal of gingival and periodontal health of the left lower incisor.

**Figure 3:**
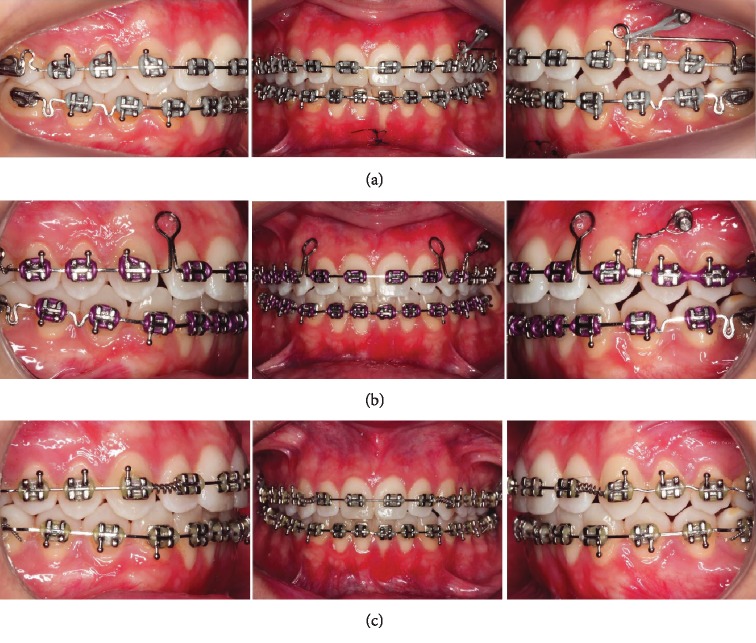
Treatment progress. (a) Distal movement of maxillary left molars supported by mini-implant and plastic surgery in the lower lip frena. (b) Retraction of the maxillary incisors. (c) Finishing arches and lingual root torque in the left mandibular central incisor.

Full fixed preadjusted appliances with 0.022 in × 0.028 in slots were placed in both arches (Figure 3). Alignment and leveling were done using NiTi wires (0.014 in and 0.016 in) followed by 0.019 in × 0.025 in SS with individualized lingual root torque in the left central incisor, and at that stage, the plasty was performed on the lower lip frena (Figure 3(a)). A mini-implant was placed between the premolars for distal movement of the posterior teeth, which was obtained by means of a sliding-jig, nickel-titanium spring and elastic chains (Figure 3(b)). A retraction arch with helical loops positioned distally to the lateral incisors (Figure 3(c)) was used.

This was followed with coordinated maxillary and mandibular finishing arches, with the maintenance of root lingual torque in the left lower central incisor (Figure 3(c)). Class II intermaxillary and vertical elastics were used throughout the active treatment.

After 20 months, the fixed appliance was removed and the superior wraparound-type retention and a fixed canine-to-canine retention were bonded.

## 6. Treatment Results

**Figure 4:**
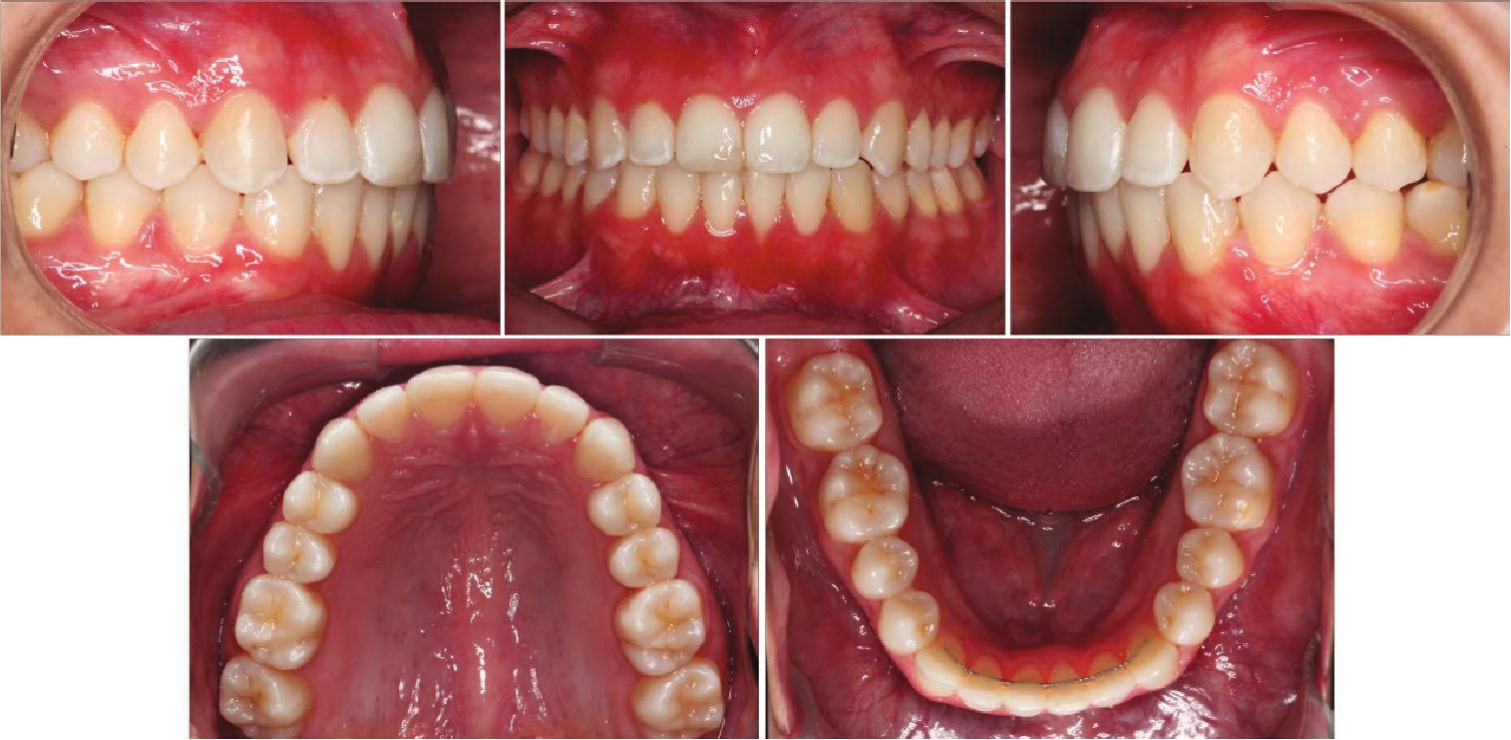
Posttreatment intraoral photographs.

**Figure 5:**
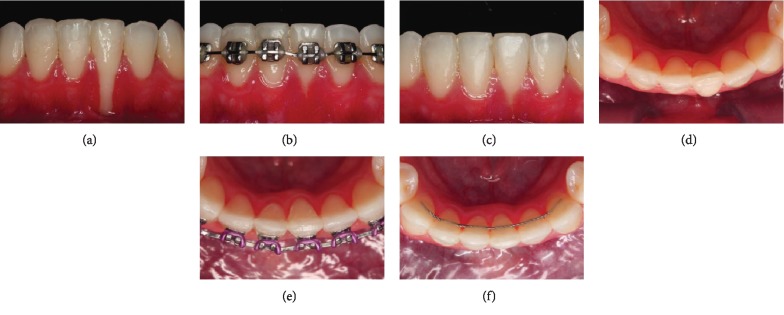
Magnitude of the gingival recession. (a, d) Initial; (b, e) progress; (c, f) final.

Complementary examinations showed that the treatment goals were achieved, with maintenance of right molar and canine Class I and Class I relationship of molar and canine on the left side, as well as normal overjet and overbite (Figure 4). There was a clinically significant improvement of the gingival recession (Figures 5(c) and 5(f)), and the prognosis for posttreatment gingival graft became much more favorable (Figures 5(a), 5(b), 5(d), and 5(e)).

**Figure 6:**
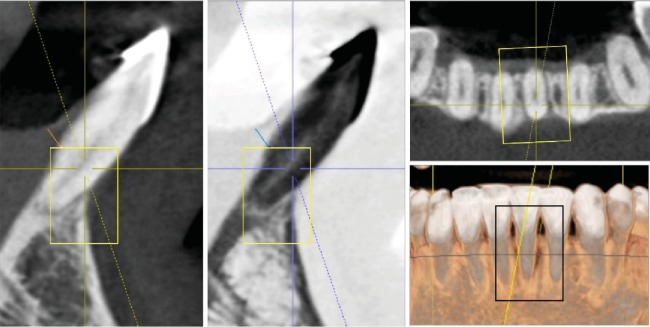
Final CBCT of the left mandibular central incisor. Sagittal, coronal, and axial views.

**Figure 7:**
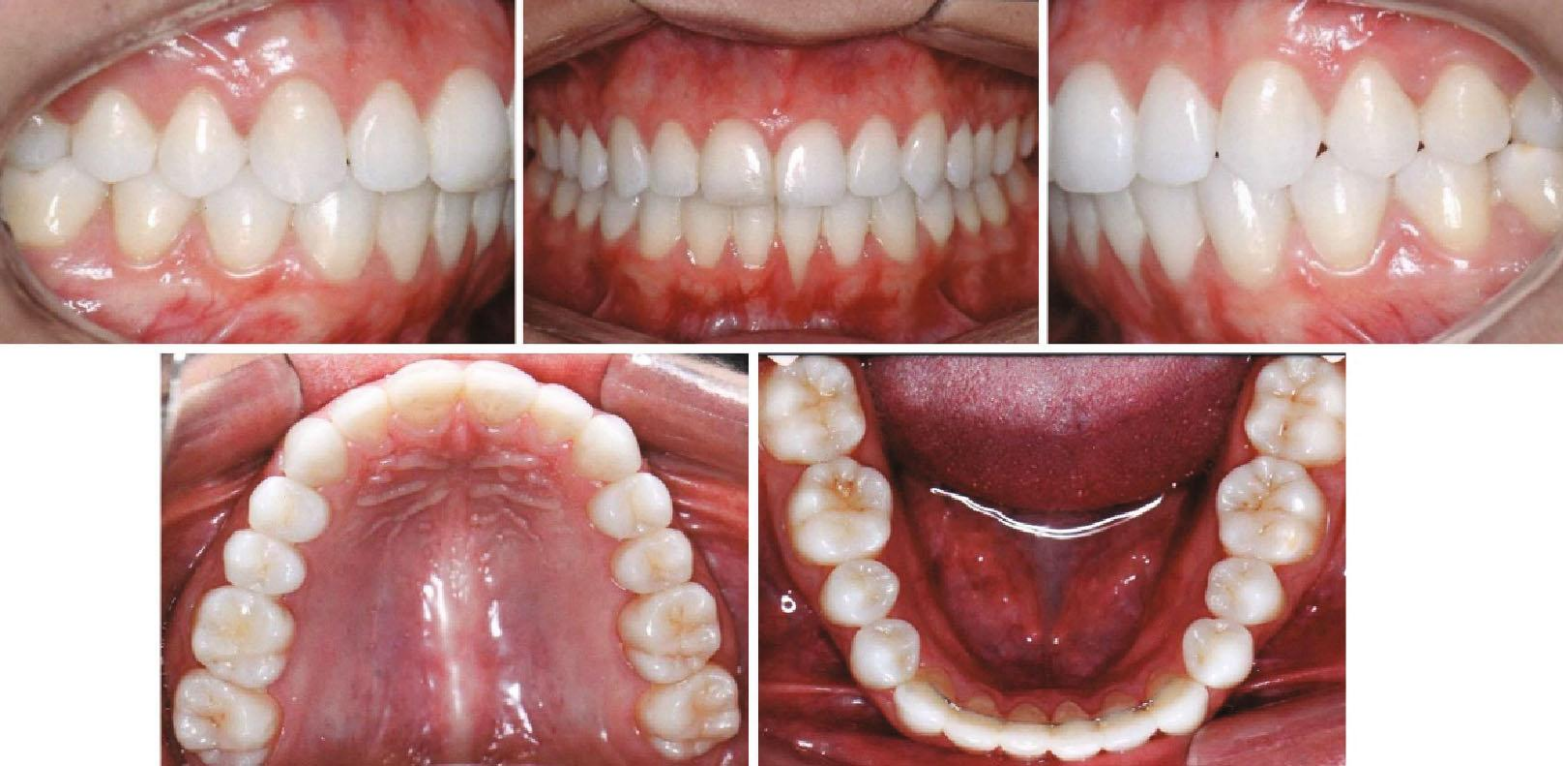
Follow-up intraoral photographs.

Tomography revealed in the axial, sagittal, and coronal views a significant change in the facial apical third of the root of the left mandibular incisor with bone formation. There were no signs of root resorption or bone loss near the mandibular incisors (Figure 6). Retention at the 1-year follow-up intraoral photos showed stable gingival health within normal limits and an improved occlusal intercuspation (Figure 7).

## 7. Discussion

The etiology of gingival recession is multifactorial. However, researchers have concluded that if orthodontic treatment is performed inaccurately, it can lead to gingival recession [4]. The patient related that she did not have gingival recession before her previous orthodontic treatment. Thus, the etiology of the gingival recession in our patient could be related to plaque accumulation or toothbrush trauma and to previous orthodontic treatment with subsequent placement of the mandibular incisor roots outside the alveolar bone, as in a similar case reported by Machado et al. [6].

The radicular exposure resulting from the gingival recession, besides the esthetic impairment, poses multiple problems for the individual, such as the occurrence of radicular hypersensitivity, difficulty in brushing in the affected region with the ease of biofilm retention, and greater susceptibility to developing root caries [9].

In the illustrated clinical case, a third-order bend inserted in a differentiated manner was used for the correction of sagittal positioning of a left lower central incisor recession.

According to Sifakakis et al., unexpected movements of the mandibular incisors after treatment can have many causes. Some movements may be provoked either by an active component in the retainer caused by the clinician during construction or bonding or by the masticatory forces deforming the wire [10].

Machado et al. [6] used a continuous archwire to move an incisor root lingually to reduce a moderate recession, while Farret et al. [11] chose a segmented titanium-molybdenum arch rather for precise measurement of the force than a continuous arch for complete control of the force used to torque the root.

In the present case, the treatment of the recession was performed by doing individual torque of the recessive teeth, followed by frenectomy. After the orthodontic retreatment and placement of the tooth within the bony housing, the patient was referred for a gingival graft, but she did not undergo the procedure because she was satisfied with the gingival health.

The absence of periodontal pockets was verified by probing after treatment and at 1 year after treatment, also increasing the good prognosis in the long term. This periodontal result after treatment may also result from good gingival width on the buccal surface of the mandibular anterior teeth and good hygiene, without inflammation during and after treatment.

The spontaneous improvement of root coverage in this patient was in contrast with Deng et al. [12] who stated that gingival recession was not reversible. Farret et al. [11] showed that at the end of the treatment, even without a graft positioned over the root, an excellent gingival pattern was obtained, with a minor recession on the facial surface not accompanied by inflammation.

The sagittal tomographic images in the occlusal view showed bone formation into the bone defect in the apex region, probably caused by the movement of the root through the cortical bone that carried the cortical wall lingually in the mandibular central incisor, thus improving the prognosis of the tooth, but Garlock et al. stated that although pretreatment cortical bone thickness, ridge width thickness, and specific tooth movements all play roles in what happens to the bone during treatment, incisor inclination was not correlated with alveolar bone height changes [13].

Orthodontic movement is not a determining factor to result in gingival recession, but when associated with thin gingiva, thin bone board, dehiscence, bone fenestration, direction of orthodontic movement, presence of inflammation, or trauma of brushing, it creates a favorable environment for the development of gingival recession. Tanaka et al. successfully advocated the grafting of subepithelial connective tissue of the palate prior to orthodontic movement, restoring the clinical crown (from 9.0 mm to 5.0 mm) on the vestibular face of the right lower central incisor [9], and most recently, Sculean and Allen presented a substantial recession coverage with laterally closed tunnel technique for the treatment of deep isolated mandibular recession [14].

The main purpose of the preadjustable bracket system is to minimize the need to introduce folds in the orthodontic wires. However, even using a preset apparatus, in certain cases, it is necessary to insert third-order folds (torque) into the rectangular arch to achieve a good orthodontic finish [15].

Antelo et al. used a versatile bracket on the lateral incisor that was bonded in an inverted way, with a rotation of 180° to express a negative torque of –10° in a clinical case with the maxillary lateral incisor for palatine and in anterior crossbite, and biomechanics was efficiently expressed without the insertion of torque in the orthodontic arch [16], and this inversion of the bracket could have been applied in the present clinical case.

Therefore, even with the use of preadjusted brackets and versatility feature, the professional must have biomechanics knowledge and technical ability with a clinical capacity of an interdisciplinary team, composed of the orthodontist, periodontist, and patient commitment.

## 8. Conclusions

The orthodontic treatment of a young woman, with severe gingival recession of the left lower central incisor, was carried out, with the application of a localized biomechanics system of forces that delivered good dental and functional occlusion and, mainly, gingival and periodontal health. The 1-year follow-up showed stable results with the periodontium within normal limits and an improved occlusal interdigitation.
